# The cellular and mutational origins of IDH mutant glioma

**DOI:** 10.1093/neuonc/noag034

**Published:** 2026-02-18

**Authors:** Benjamin J Lerman, Olivia M Doyle, Joseph F Costello

**Affiliations:** Department of Pediatrics, University of California San Francisco, San Francisco, California, USA; Department of Neurological Surgery, University of California San Francisco, San Francisco, California, USA; Department of Neurological Surgery, University of California San Francisco, San Francisco, California, USA

IDH mutant diffuse glioma typically presents as a sizeable mass composed of billions of cells, yet they are slow growing with few if any mitoses. This has led to speculation that the tumor originates years or even decades before diagnosis. Without access to tissue at these formative stages, it is therefore quite a challenge to trace far back in the tumor’s evolution to discover the mutational and cellular origins. In the January 8, 2026 edition of *Science*, Park, Lee, and colleagues used strategic sampling within and outside of the patient’s tumor mass, along with careful analysis of an IDH mutation-driven mouse model to address this dual challenge.^1^

Understanding tumor cellular and genomic origins is key to developing better tumor models and for the development and testing of therapies that will be effective and durable. While prior studies in tumor tissue inferred that IDH is the initial oncogenic hit, conclusive evidence has been elusive.^2–5^ Park et al. provide direct evidence that the IDH mutation is the initial driver mutation and that it arises in the glial progenitor cell lineage (GPC).

Park et al. collected 142 samples from 70 individuals: 32 with IDH mutant tumors and 38 IDH wild-type controls. Because actively proliferating progenitor cells have an increased risk of somatic mutation, Park et al. collected samples not only from within the tumor mass but also from the peritumoral cortex and the subventricular zone (SVZ). Although neural stem cells in the SVZ acquire the initial driver mutations in IDH wild-type glioblastoma, it was unknown whether the cell lineage or brain region of origin would differ in IDH mutant tumors.^6^ In approximately one-third of samples taken from the peritumoral cortex, IDH mutation was identified at ultra-low levels and in the absence of other drivers present in the tumor mass from the same patient. However, a small number of somatic passengers were also shared between peritumoral cortex and the tumor, suggesting the presence of a clonal population of premalignant cells containing IDH mutation as the sole driver. Notably, these cells were not detected in all ten samples from the SVZ, a neurogenic zone and region where this laboratory previously identified TERT promoter mutant pre-tumor cells in GBM patients.^6^

To investigate the cell population in the peritumoral cortex harboring the IDH mutation—the elusive “cell lineage of origin”—the authors then sorted nuclei by canonical neuronal (NeuN) and oligodendrocyte (OLIG2) markers. The IDH mutation was present in OLIG2+/NeuN- and OLIG2-/NeuN- samples but absent in cells expressing the neuronal marker, suggesting that the cell of origin is a member of the glial rather than neuronal lineage. After spatial transcriptomics identified oligodendrocyte precursor cells (OPCs) as the predominant non-neoplastic, IDH mutant-expressing cell type, the tumorigenic potential of mouse OPC equivalents was assessed using a Cre–lox-based genetic engineering system. They selectively introduced the IDH mutation into cells expressing Ng2—a marker of OPCs, as well as a small subset of astroglial progenitor cells. While IDH mutant OPCs exhibited enhanced proliferation compared to IDH wild-type OPCs, they did not form tumors. Only with the CRISPR-mediated knockout of TP53, ATRX, and NF1 did IDH mutant OPC develop into glioma *in vivo* and exhibit a transcriptional profile resembling patient-derived IDH mutant gliomas. To determine if only progenitor cells and not mature oligodendrocytes were capable of malignant transformation, the authors introduced identical genetic changes into mature oligodendrocytes and astrocytes, which did not form tumors.

In directly identifying IDH as the initial mutation and proposing GPCs as the putative lineage of origin in IDH mutant glioma, Park et al. take critical steps towards resolving these fundamental questions surrounding gliomagenesis. This refined understanding of the sequence of tumor-initiating molecular events and cell population should lead to the development of more accurate models of glioma in which to test novel therapeutics. Furthermore, the identification of IDH mutant-containing cells in radiographically and histologically normal-appearing peritumoral regions raises important clinical questions for tumor prevention, detection, treatment, and surveillance. What is the incidence of IDH mutation in GPC in the general population? What is the rate of malignant transformation of these cells? Can they be identified and eliminated, or held in check, before transformation occurs? Mutation-selective inhibition of IDH has emerged in recent years as a cornerstone of therapy for these patients.^7^ How do the premalignant IDH mutant cells in a glioma patient respond to such a treatment?

This study represents a major advancement in our understanding of the earliest and previously inaccessible phases of evolution of IDH mutant glioma, though knowledge gaps remain. All *in vivo* models of IDH mutant glioma to date have been limited in their generalizability by the driver alterations required to induce gliomagenesis in the mouse and by the species-specific genomic differences, which are a significant barrier for modeling TERT promoter mutant, 1p/19q co-deleted oligodendroglioma in mice. Despite the identification of the cell of origin and specific targeting of oncogenes to that population, Park et al. faced similar limitations in this study. They found that introduction of the IDH mutation into OPCs—even in combination with the canonical TP53 and ATRX alterations—was insufficient to form a tumor. The addition of NF1 knockout, which occurs in human IDH mutant glioma albeit infrequently, to the mouse OPCs was required to form tumors. Interestingly, substitution of CDKN2A knockout, which is associated with increased malignancy in human IDH mutant glioma, for NF1 did not induce tumor formation. This work should further inspire the field to work towards defining the minimal conditions and genetic alterations for successful clonal expansion and tumorigenesis and ultimately engineering new human cell-based and animal models.

## Conflict of Interest Statement

BJL, OMD, and JFC do not endorse any conflicts of interest related to this publication.
